# Prevalence and association of asthma and allergic sensitization with dietary factors in schoolchildren: data from the french six cities study

**DOI:** 10.1186/s12889-015-2320-2

**Published:** 2015-09-30

**Authors:** Danielle Saadeh, Pascale Salameh, Denis Caillaud, Denis Charpin, Frédéric De Blay, Christine Kopferschmitt, François Lavaud, Isabella Annesi-Maesano, Isabelle Baldi, Chantal Raherison

**Affiliations:** Clinical and Epidemiological Research Laboratory, Lebanese University, Hadath, Lebanon; Hôpital Gabriel Montpied, Clermont-Ferrand, France; Hôpital Nord, Marseille, France; Hôpital Civil, Strasbourg, France; Hôpital Maison Blanche, Reims, France; EPAR, UMR-S 1136, Institute Pierre Louis of Epidemiology and Public Health, INSERM and UPMC Sorbonne Universities, Paris, France; INSERM U897, Institut de Santé Publique d’Epidémiologie et de Développement, Laboratoire Santé Travail Environnement, Université de Bordeaux, Bordeaux, France; Service des Maladies Respiratoires, Hôpital du Haut-Lévèque, Avenue de Magellan, Pessac, France

**Keywords:** Allergic sensitization, Asthma, Children, Dietary factors, French Six Cities Study, ISAAC

## Abstract

**Background:**

The prevalence of asthma and allergy has recently risen among children. This increase in prevalence might be related to various factors, particularly diet. The aim of this study is to assess the prevalence and association of asthma and allergic sensitization with dietary factors in the French Six Cities Study.

**Methods:**

Cross-sectional studies were performed among 7432 schoolchildren aged 9–11 years in Bordeaux, Clermont-Ferrand, Créteil, Marseille, Reims, and Strasbourg. Parental questionnaires, based on the International Study on Asthma and Allergies in Childhood (ISAAC), were used to collect information on allergic diseases and potential exposure factors including a food frequency questionnaire to evaluate dietary habits. Skin prick testing to common allergens for allergic sensitization and bronchial hyper-responsiveness (BHR) testing to exercise were performed. Confounders control was performed with multiple logistic regressions.

**Results:**

Asthma symptoms, asthma and allergic sensitization were more prevalent in boys than in girls and were more prevalent in the South than in the North of France. After adjustment for confounders, fruit juice intake was associated with a low prevalence of lifetime asthma (ORa [95 % CI]; 0.73 [0.56–0.97]), butter intake was positively associated with atopic wheeze (1.48 [1.07–2.05]) and having lunch at the canteen 1–2 times/week compared to never or occasionally was associated with a lower prevalence of past year wheeze (0.71 [0.52–0.96]), lifetime asthma (0.76 [0.60–0.96]) and allergic sensitization (0.80 [0.67–0.95]). Meat intake was inversely related to past year wheeze among atopic children (0.68 [0.50–0.98]) while fast food consumption and butter intake were associated with an increase prevalence of asthma (2.39 [1.47–3.93] and 1.51 [1.17–2.00] respectively). Fish intake was associated with a lower prevalence of asthma among non-atopic children (0.61 [0.43–0.87]. None of the dietary factors was associated with BHR.

**Conclusions:**

Diet is associated with wheeze, asthma and allergic sensitization but not with BHR in children. These results provide further evidence that adherence to a healthy diet including fruits, meat and fish seems to have a protective effect on asthma and allergy in childhood. However, prospective and experimental studies are needed to provide causal evidence concerning the effect of diet on asthma and atopy.

## Background

The prevalence of asthma and allergic diseases among children and adolescents has rapidly increased over the last 10 years, especially in the Western world [1–4], and it has become a serious public health issue [5]. Unfortunately, the reasons for this increase are still obscure but risk factors including genetic predisposition and environmental factors (change in lifestyle, air pollution, etc.) could be responsible [6, 7]. In fact, one hypothesis to explain the occurrence of asthma and allergies in childhood, are related to lifestyle factors. Among these factors, dietary habits may be one of the contributing factors [8, 9].

**Fig. 1:**
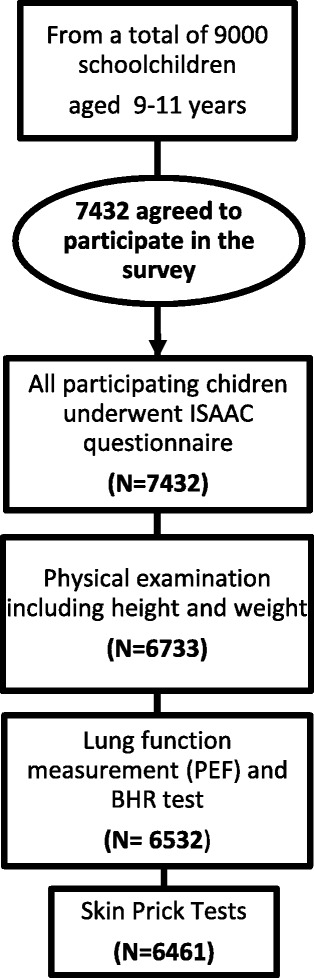
Flow chart of participants. ISAAC, International Study of Asthma and Allergies in Childhood; PEF, Peak Expiratory Flow; BHR, Bronchial Hyper-responsiveness

Several studies have shown that some food could increase the risk of asthma and allergies [10–12], while others hypothesize that other food products could have a protective effect on allergies, such as vegetables [13–15], and oily fish [11]. Dietary fat intake was shown to have an association with atopy in children. Therefore, high margarine consumption was positively associated with hay fever, while butter consumption was negatively associated with hay fever [16]. Furthermore, protein-rich and fat-rich foods of animal origin were associated with a higher prevalence of asthma [17, 18]. Special attention was given to n-3-poly unsaturated fatty acids (PUFA) intake. In fact, there is evidence that fish rich in n-3-PUFA has anti-inflammatory properties and may modulate the immune response towards a Th1 type [19]. In fact, fish consumption was shown to have a protective effect on childhood asthma in several studies [20–22]. Moreover, in the ISAAC, ecological studies suggested a relationship between the intake of trans-fatty acids and the prevalence of childhood asthma and allergies [23], and a consistent inverse relationship was seen between prevalence rates of asthma, allergic rhinitis and eczema and the intake of vegetables [24]. Furthermore, decreased intake of antioxidants found mostly in fresh fruit and vegetables, has been shown to have a role in the development of atopy [19, 24, 25].

From the methodological point of view, the relationship between diet and health can be assessed at the level of food studied independently from each other. However, we hypothesize that nutrition is a general complex habit, and that the association of food products may differ from one individual to another with regard to their effect on health [26, 27].

In this study, we aim to assess, the prevalence and association of asthma, asthma symptoms, allergic sensitization and bronchial hyper-responsiveness (BHR) with dietary factors in a large French population-based sample of schoolchildren.

## Methods

### Study design and study population

The data set is part of the French Six Cities Study. Cross-sectional studies were conducted, in 2000–2001, in 6 French ISAAC centers: Bordeaux, Clermont-Ferrand, Créteil, Marseille, Reims, and Strasbourg. Among a total of 9000 children aged 9–11 years old and in 4th and 5th grade, 7432 agreed to participate in the French six cities survey. We classified their place of residence according to the study centers divided into North (Créteil, Reims, and Strasbourg) and South (Bordeaux, Clermont-Ferrand and Marseille) of France. Figure 1 show the flow chart of participants with the number of children who underwent ISAAC questionnaire, physical examination, lung function measurements, BHR test and Skin Prick Tests.

### Questionnaire

Standardized self-administered epidemiological questionnaires were developed on demographics, wheezing, asthma, allergic rhinitis (AR) and atopic eczema. The main questions were derived from the International Study of Asthma and Allergies in Childhood (ISAAC) questionnaire [28]. These included detailed questions on the occurrence and severity of atopic symptoms (asthma, allergic rhinitis, eczema) and their potential exposure factors. Such questions had been previously validated and translated from English into French by a native French speaker, then back-translated into English by a native English speaker. All questionnaires were completed by the parents.

### Dietary assessment

Children’s feeding dietary habits were collected by a food frequency questionnaire (FFQ). The FFQ included the following food items and dietary factors commonly consumed in France: soft drinks, fruit juice, cooked vegetables, raw vegetables, citrus fruit, red meat, white fish, dairy products, fast food, and canteen lunch. The FFQ was divided by frequency of consumption per week (never/occasionally, 1–2 times, ≥3 times). In addition a question was added, independently from the FFQ, to assess the most consumable fatty acids by children (butter, margarine and vegetable oil).

### Clinical tests

The children’s consent was obtained before conducting clinical examination in their classrooms. Then a physician conducted a physical examination including data on respiratory symptoms, skin prick tests, lung function measurements and BHR testing. Furthermore, data on height and weight were obtained for 6733 children. Fieldworkers were trained to conduct these respiratory function tests and physical examinations.

### Skin-prick test (SPT)

SPTs for atopy were performed on 6461 children using Stallerpoints (Stallergènes Laboratoires, Antony, France). The skin tests were performed by the SPT technique according to the ISAAC protocol [29]. Children were tested for the following common food and aeroallergens: *Dermatophagoides pteronyssinus, Dermatophagoides farinaee,* cat fur*, Alternaria tenius,* mixed grass and tree pollens, peanut, codfish, *Blatta germanica* and egg. At least one positive reaction (weal size at least 3 mm and greater than the negative control) was defined as a positive SPT, therefore having an allergic sensitization.

### Bronchial hyper-responsiveness (BHR) test

BHR was assessed according to the standardized protocol of the run test [30–32]. Baseline peak expiratory flow (PEF) was measured in 6532 children who agreed. Post-exercise PEF was recorded immediately after the challenge, five minutes, ten minutes and fifteen minutes later. A child was considered to have BHR to exercise if the decrease in PEF after exercise exceeded 10 %. Subsequently, if a fall in PEF of 10 % was determined or if the child presented any respiratory symptom, he was first examined by the physician and a β2-agonist with inhalation chamber was administered in order to ensure the reversibility of the bronchospasm.

### Health outcomes

The following health outcomes were considered in the analysis: *Past year wheeze* (a history of “chest wheezing or whistling in the chest over the past 12 months” (Yes/No)) used as an indicator of childhood asthma; *Atopic wheeze* was defined as wheeze during the past year and a positive SPT; *Lifetime asthma* (a history of asthma at some point in life according to the standardized question “Has your child ever had asthma?” (Yes/No)) used to investigate the lifetime prevalence of asthma; A*topy* in children was defined as having an allergic sensitization as described above in the SPT paragraph; and *BHR* as described in the paragraph above.

### Statistical analysis

All continuous variables are presented as mean (m) and standard deviation (SD), and the categorical variables are presented as frequencies. Correlations between dietary factors and health outcomes were determined by the Pearson chi-square test.

Multiple logistic regressions models were used to assess the association between dietary factors that were statistically significant in the univariate analysis (p ≤ 0.2) as independent variables and all investigated health outcomes as dependent variables. Hosmer and Lemeshow test was calculated for the evaluation of the model’s goodness-of-fit. The relative risks of asthma related symptoms and allergic sensitization based on all dietary factors were estimated by calculating the adjusted odds ratios (ORa) and corresponding 95 % confidence intervals (95 % CI). ORs were adjusted for potential confounders to control for increased risk of atopy.

The following potential confounders were considered: gender, place of residence (North or South of France according to ISAAC centers), parental atopic disease (defined by whether the father or the mother of the child had ever suffered from asthma, AR or eczema), number of siblings (0, 1–2, ≥3), maternal education, parental ethnic origins, breastfeeding, day care center or nursery, overweight and obesity and current exposure to environmental tobacco smoke (ETS) (“Does anybody at present smoke inside your child’s home?” Yes/No).

Differences between strata were assessed by z-test. Depending on prevalence and completeness of variables, the numbers of subjects in the models differ.

All reported probability values (*p-*values) were based on two-sided tests and a *p* < 0.05 was considered statistically significant. All analyses were performed using the Statistical Package for Social Science (SPSS) version 21.

## Results

### Descriptive results

The figure illustrates the flow of participants with the number of children who underwent ISAAC questionnaire, data of physical examination, SPT, the lung function measurements and BHR test. About 82 % of the children aged 9–11 years (7432/9000) agreed to participate in the study. Among these children, 21 % were from the Créteil ISAAC center, 18 % from Bordeaux, 16 % from Clermont-Ferrand, 16 % from Marseille, 16 % from Strasbourg and 13 % from Reims. Characteristics of the children who participated in the study are shown in Table 1. About 48 % of these children were breast-fed, 64 % of them were breast-fed for less than four months and 36 % were breast-fed for four months or more. About 30 % of the children were in a day care center or nursery for at least six months in their infancy.

**Table 1:** Characteristics of the children who underwent clinical examination and their parents who completed the questionnaire (*n* = 7432)

Characteristics	Child	Mother	Father
Age, years (m ± SD)		38.4 ± 5.1	41.4 ± 6.3
9 years (%)	27.8		
10 years (%)	48.7		
11 years (%)	23.5		
Gender (male, %)	49.4		
Place of residence (%)			
North of France	49.9		
South of France	50.1		
Weight in Kg (m ± SD)	36.1 ± 8.3		
Height in meter (m ± SD)	1.4 ± 0.1		
Overweight and obesity (%)	21.0		
Current ETS (%)	40.0		
Parental atopic disease (%)	39.5		
Ethnic origins (%)^a^			
Metropolitan France		66.4	60.8
French overseas departments		3.2	2.1
Southern Europe		3.5	3.6
Northern Africa		7.6	8.3
Sub-Saharan Africa		2.5	2.6
Asia		3.1	3.1
Other		3.0	2.9
Education (%)^a^			
Primary		9.8	9.4
Secondary		37.5	32.3
High school and university		31.7	30.1
Other		4.3	4.2
Number of siblings (%)			
No siblings	47.9		
1-2 siblings	46.1		
≥ 3 siblings	6.0		

*ETS* Exposure to Tobacco Smoke

^a^Totals do not sum to the sample size due to missing data

### Prevalence of asthma symptoms, asthma and allergic sensitization

The prevalence of health outcomes assessed in this study was as follows: 3.5 % for atopic wheeze; 6.7 % for past year wheeze; and 8.9 % for lifetime asthma. The prevalence of BHR to exercise was 7.9 %.

Among the 6461 children who underwent SPT, 27.5 % had at least one positive test and were considered as having allergic sensitization (sensitized children); 11.3 % of them were mono-sensitized (had only one positive SPT) and 16.2 % were poly-sensitized (had two or more positive SPTs). The most prevalent allergen SPT positivity in decreasing order was as follows: *Dermatophagoides pteronyssinus* (17 %), *Dermatophagoides farinaee* (11.4 %), grass pollen (9.5 %), mixed tree (3.8 %), cat fur (3.9 %) and *Alternaria tenius* (2.8 %).

After adjustment for confounders in logistic regression models, boys were significantly more affected than girls by atopic wheeze (ORa 0.58, 95 % CI [0.42–0.80]), past year wheeze (ORa 0.74, 95 % CI [0.57–0.96]) lifetime asthma (ORa 0.56, 95 % CI [0.45–0.69]) and allergic sensitization (ORa 0.68, 95 % CI [0.59–0.79]), but not with BHR. We also found significant differences between Northern and Southern cities of France and prevalence of asthma symptoms, allergic sensitization and BHR. Therefore, atopic wheeze, allergic sensitization and BHR were more prevalent in the South than in the North (ORa 1.46, 95 % CI [1.05–2.04]; ORa 1.53, 95 % CI [1.32–1.78] and ORa 1.53, 95 % CI [1.20–1.94] respectively).

### Dietary factors and allergic diseases

Frequencies of consumption of the dietary factors included in the FFQ are shown in Table 2. Concerning fatty acids intake, 29 % of children consumed butter, 64 % vegetable oil and 28 % margarine. The majority of school children (93 %) never or occasionally consumed fast food. In addition, about 46 % of the children always ate their lunch at the canteen, 21 % ate from time to time at the canteen and 33 % never ate at the canteen.

**Table 2:** Consumption frequency of the dietary factors included in the FFQ (*n* = 7432)

Dietary factors	Number	Percent
Soft drinks intake		
Never/occasionally	3781	50.9
1–2 times/week	1909	25.7
≥ 3 times/week	1742	23.4
Fruit juice intake		
Never/occasionally	2654	35.7
1–2 times/week	1515	20.4
≥ 3 times/week	3263	43.9
Cooked vegetables intake		
Never/occasionally	1451	19.5
1–2 times/week	1639	22.1
≥ 3 times/week	4342	58.4
Raw vegetables intake		
Never/occasionally	2258	30.4
1–2 times/week	2062	27.7
≥ 3 times/week	3112	41.9
Citrus fruit intake		
Never/occasionally	3087	41.5
1–2 times/week	2095	28.2
≥ 3 times/week	2250	30.3
Red meat intake		
Never/occasionally	1738	23.4
1–2 times/week	2796	37.6
≥ 3 times/week	2898	39.9
Fish intake		
Never/occasionally	5695	76.6
1–2 times/week	1453	19.6
≥3 times/week	284	3.8
Dairy products intake		
Never/occasionally	1085	14.6
1–2 times/week	323	4.3
≥ 3 times/week	6024	81.1
Fast food consumption (*N* = 6515)		
Never/occasionally	6065	93.1
1–2 times/week	413	6.3
≥ 3 times/week	37	0.6
Canteen lunch (*N* = 6790)		
Never/occasionally	2211	32.6
1–2 times/week	1443	21.3
≥ 3 times/week	3136	46.2

Our results showed that butter intake was associated positively with past year wheeze (ORa 1.24, 95 % CI [1.01–1.54]) and lifetime asthma (ORa 1.24, 95 % CI [1.04–1.49]) while consumption of vegetable oil was positively associated with atopic wheeze (ORa 1.56, 95 % CI [1.16–2.11]).

Table 3 shows the associations between dietary factors and atopic wheeze, past year wheeze, lifetime asthma, SPT positivity and BHR. Atopic wheeze was negatively associated with fruit juice consumption equal or more than 3 times per week compared to none or occasional consumption (ORa 0.66, 95 % CI [0.43–0.91]; *p* = 0.007) and compared to none or occasional eating at the canteen, canteen lunch 1 to 2 times per week was as well negatively associated with atopic wheeze (ORa 0.57, 95 % CI [0.37–0.88]). Furthermore, consumption of red meat 1–2 times/week vs. never or occasionally was found to be positively associated with atopic wheeze (ORa 1.35, 95 % CI [1.05–1.68]; *p* = 0.022). Consumption of white fish 1–2 times/week vs. never or occasionally was negatively correlated with past year wheeze (ORa 0.75, 95 % CI [0.53–0.93]; *p* = 0.028), while we found no significant associations between fish intake and SPT positivity and BHR. In addition, we found that compared to none or occasional consumption, fruit juice intake ≥ 3 times per week was protective for lifetime asthma (ORa 0.73, 95 % CI [0.55–0.96]). Canteen lunch 1–2 times/week vs. never or occasionally was negatively associated with past year wheeze (ORa 0.72, 95 % CI [0.52–0.99]; *p* = 0.014) and SPT positivity (ORa 0.83, 95 % CI [0.69–0.99]; *p* = 0.001). Cooked vegetable intake ≥ 3 times per week vs. never or occasionally and canteen lunch 1–2 times/week vs. never or occasionally were negatively associated with BHR (ORa 0.77, 95 % CI [0.57–0.94]; *p* = 0.009 and ORa 0.82, 95 % CI [0.71–0.91]; *p* = 0.005 respectively).

**Table 3:** Association between dietary factors and atopic wheeze, past year wheeze, lifetime asthma, SPT positivity and BHR

	Atopic wheeze^a^		Past year wheeze^a^		Lifetime asthma^a^		SPT positivity^a^		BHR^a^	
	OR	95 % CI	OR	95 % CI	OR	95 % CI	OR	95 % CI	OR	95 % CI
Soft drink intake (*N*)	5200		2322		6089		5902		5954	
Never/occasionally	1	Ref	1	Ref	1	Ref	1	Ref	1	Ref
1–2 times/week	1.05	0.70 to 1.58	1.00	0.72 to 1.38	1.07	0.82 to 1.40	1.18	0.98 to 1.43	1.10	0.82 to 1.47
≥ 3 times/week	0.89	0.57 to 1.39	0.88	0.62 to 1.26	1.03	0.77 to 1.37	0.99	0.81 to 1.22	0.94	0.68 to 1.30
*p-value*		0.839		0.562		0.615		0.062		0.192
Fruit juice intake (*N*)	5200		2322		6089		5902		5954	
Never/occasionally	1	Ref	1	Ref	1	Ref	1	Ref	1	Ref
1–2 times/week	0.76	0.53 to 1.09	0.97	0.72 to 1.30	1.02	0.81 to 1.29	0.94	0.79 to 1.11	1.01	0.77 to 1.31
≥ 3 times/week	0.66	0.43 to 0.91	0.89	0.64 to 1.24	0.73	0.55 to 0.96	0.96	0.79 to 1.15	1.15	0.87 to 1.53
*p-value*		0.007		0.275		0.054		0.492		0.793
Cooked vegetable intake (*N*)	5200		2322		6089		5902		5954	
Never/occasionally	1	Ref	1	Ref	1	Ref	1	Ref	1	Ref
1–2 times/week	0.99	0.55 to 1.79	1.19	0.76 to 1.87	1.02	0.70 to 1.49	0.88	0.66 to 1.16	1.06	0.71 to 1.59
≥ 3 times/week	0.98	0.66 to 1.45	0.94	0.68 to 1.29	1.10	0.86 to 1.40	1.05	0.88 to 1.26	0.77	0.57 to 0.94
*p-value*		0.078		0.170		0.102		0.775		0.009
Raw vegetable intake (*N*)	5200		2322		6089		5902		5954	
Never/occasionally	1	Ref	1	Ref	1	Ref	1	Ref	1	Ref
1–2 times/week	1.05	0.68 to 1.63	0.98	0.69 to 1.40	0.94	0.70 to 1.25	0.99	0.81 to 1.21	1.05	0.76 to 1.44
≥ 3 times/week	1.07	0.75 to 1.54	1.05	0.78 to 1.41	1.11	0.87 to 1.40	1.03	0.87 to 1.22	1.15	0.88 to 1.48
*p-value*		0.094		0.682		0.532		0.428		0.645
Fruit intake (*N*)	5200		2322		6089		5902		5954	
Never/occasionally	1	Ref	1	Ref	1	Ref	1	Ref	1	Ref
1–2 times/week	1.06	0.72 to 1.56	1.15	0.84 to 1.58	1.10	0.85 to 1.41	0.98	0.82 to 1.17	1.20	0.90 to 1.59
≥ 3 times/week	1.06	0.71 to 1.57	1.17	0.85 to 1.62	0.99	0.77 to 1.29	1.11	0.93 to 1.33	1.31	0.99 to 1.74
*p-value*		0.109		0.670		0.351		0.592		0.468
Red meat intake (N)	5200		2322		6089		5902		5954	
Never/occasionally	1	Ref	1	Ref	1	Ref	1	Ref	1	Ref
1–2 times/week	1.35	1.05 to 1.68	1.03	0.69 to 1.52	0.84	0.60 to 1.18	0.94	0.74 to 1.19	1.26	0.89 to 1.80
≥ 3 times/week	0.83	0.59 to 1.16	0,89	0.68 to 1.18	0.93	0.75 to 1.16	0.96	0.83 to 1.12	1.25	0.98 to 1.59
*p-value*		0.022		0.587		0.261		0.546		0.074
Fish intake (*N*)	5200		2322		6089		5902		5954	
Never/occasionally	1	Ref	1	Ref	1	Ref	1	Ref	1	Ref
1–2 times/week	2.78	0.66 to 11.61	0.75	0.53 to 0.93	1.11	0.61 to 2.03	1.05	0.69 to 1.61	1.44	0.69 to 3.02
≥ 3 times/week	2.46	0.57 to 10.51	1.25	0.54 to 2.89	1.22	0.65 to 2.26	1.03	0.66 to 1.60	1.67	0.78 to 3.57
*p-value*		0.042		0.028		0.789		0.882		0.524
Dairy product intake (*N*)	5200		2322		6089		5902		5954	
Never/occasionally	1	Ref	1	Ref	1	Ref	1	Ref	1	Ref
1–2 times/week	0.66	0.23 to 1.84	0.79	0.40 to 1.56	1.06	0.62 to 1.82	1.03	0.70 to 1.51	0.64	0.32 to 1.28
≥ 3 times/week	0.70	0.28 to 1.78	0.72	0.37 to 1.38	0.99	0.58 to 1.69	0.70	0.46 to 1.05	0.97	0.54 to 1.74
*p-value*		<0.001		0.186		0.985		0.428		0.271
Fast food consumption (*N*)	4635		2198		5809		5165		5211	
Never/occasionally	1	Ref	1	Ref	1	Ref	1	Ref	1	Ref
1–2 times/week	0.36	0.07 to 1.86	1.68	0.17 to 15.92	1.27	0.15 to 10.21	3.46	0.43 to 27.52	0.85	0.10 to 6.82
≥ 3 times/week	0.53	0.09 to 2.95	1.96	0.19 to 19.38	1.47	0.17 to 12.18	2.89	0.35 to 23.52	0.92	0.11 to 7.75
*p-value*		0.715		0.643		0.302		0.407		0.079
Canteen lunch (N)	4817		2300		6046		5371		5414	
Never/occasionally	1	Ref	1	Ref	1	Ref	1	Ref	1	Ref
1–2 times/week	0.57	0.37 to 0.88	0.72	0.52 to 0.99	0.80	0.62 to 1.03	0.83	0.69 to 0.99	0.82	0.71 to 0.91
≥ 3 times/week	0.93	0.64 to 1.37	0.88	0.63 to 1.22	0.78	0.60 to 1.03	0.88	0.73 to 1.06	1.07	0.81 to 1.42
*p-value*		<0.001		0.014		0.011		<0.001		0.005

*SPT* Skin Prick Test, *BHR* Bronchial hyper-responsiveness

^a^Adjusted for gender, place of residence, parental atopic disease, number of siblings (0, 1–2, ≥3), maternal education, parental ethnic origins, breastfeeding, day care center or nursery, overweight and obesity and current exposure to environmental tobacco smoke (ETS)

After adjustment for the following confounders: gender, place of residence, number of siblings, parental ethnic origins, maternal education, parental atopic disease, breastfeeding, day care center or nursery, overweight and obesity and current exposition to Environmental Tobacco Smoke (ETS), in the logistic regression models, fruit juice intake ≥ 3 times per week compared with none or occasional consumption was still found to be protective for lifetime asthma (ORa 0.73, 95 % CI [0.56–0.97]). In contrast, butter intake was positively associated with atopic wheeze (ORa 1.48, 95 % CI [1.07–2.05]). Moreover, canteen lunch 1 to 2 times per week compared to never or occasionally was negatively associated with atopic wheeze (ORa 0.56, 95 % CI [0.36–0.85]), past year wheeze (ORa 0.71, 95 % CI [0.52–0.96]), lifetime asthma (ORa 0.76, 95 % CI [0.60–0.96]) and allergic sensitization (ORa 0.80, 95 % CI [0.67–0.95]). None of the dietary factors included in the logistic regression was associated with BHR.

Analyses performed after stratification by SPT positivity are shown in Table 4. Meat intake was inversely related to past year wheeze among atopic children (ORa 0.68, 95 % CI [0.50–0.98]). In contrast, fast food consumption, butter intake and canteen lunch were positively associated with wheeze during the past year among children with a positive SPT (ORa 2.39, 95 % CI [1.47–3.93]; ORa 1.51, 95 % CI [1.17–2.00] and ORa 1.64, 95 % CI [1.20–2.30] respectively). Therefore, among children with a negative SPT, fish intake was associated significantly with fewer wheezers (ORa 0.61, 95 % CI [0.43–0.87]).

**Table 4:** Association between dietary factors^b^ and past year wheeze according to skin prick test (SPT)

	Wheeze among SPT positive^a^		Wheeze among SPT negative^a^		Difference between strata (*p* for z-test)
	OR	95 % CI	OR	95 % CI	
Soft drink intake (*N*)	86		56		
No	1	Ref	1	Ref	
Yes	1.03	0.78 to 1.33	1.24	0.91 to 1.67	0.832
Fruit juice intake (*N*)	123		67		
No	1	Ref	1	Ref	
Yes	1.27	0.94 to 1.76	0.96	0.71 to 1.32	0.681
Cooked vegetable intake (*N*)	157		86		
No	1	Ref	1	Ref	
Yes	0.83	0.51 to 1.33	0.95	0.62 to 1.40	0.889
Raw vegetable intake (*N*)	139		77		
No	1	Ref	1	Ref	
Yes	1.15	0.84 to 1.63	1.02	0.72 to 1.49	0.793
Fruit intake (*N*)	110		57		
No	1	Ref	1	Ref	
Yes	1.08	0.82 to 1.41	0.89	0.67 to 1.18	0.980
Red meat intake (*N*)	145		83		
No	1	Ref	1	Ref	
Yes	0.68	0.50 to 0.98	1.07	0.76 to 1.55	0.459
Fish intake (*N*)	41		19		
No	1	Ref	1	Ref	
Yes	0.77	0.58 to 1.02	0.61	0.43 to 0.87	0.040
Dairy product intake (*N*)	166		91		
No	1	Ref	1	Ref	
Yes	1.31	0.62 to 5.32	1.28	0.76 to 2.41	0.366
Fast food consumption (*N*)	17		5		
No	1	Ref	1	Ref	
Yes	2.39	1.47 to 3.93	0.66	0.33 to 1.09	0.407
Canteen lunch (*N*)	65		138		
No	1	Ref	1	Ref	
Yes	1.64	1.20 to 2.30	1.01	0.78 to 1.33	0.023
Butter intake (*N*)	65		37		
No	1	Ref	1	Ref	
Yes	1.51	1.17 to 2.00	1.18	0.87 to 1.54	0.045
Vegetable oil intake (*N*)	127		69		
No	1	Ref	1	Ref	
Yes	1.22	0.90 to 1.69	1.11	0.80 to 1.64	0.303
Margarine intake (*N*)	44		22		
No	1	Ref	1	Ref	
Yes	0.67	0.51 to 0.84	0.69	0.49 to 0.95	0.041

^a^Adjusted for gender, place of residence, parental atopic disease, number of siblings (0, 1–2, ≥3), maternal education, parental ethnic origins, breastfeeding, day care center or nursery, overweight and obesity and current exposure to environmental tobacco smoke (ETS)

^b^Dietary variables in the ‘never’ and ‘less than once per week’ categories were classified as NO and dietary variables in the ‘1–2 times per week’, ‘3–6 times per week’ and ‘once per day or more often’ were classified as YES

## Discussion

This study is the first in France to assess the prevalence of asthma related symptoms and allergic sensitization and their associations with dietary factors in a large population-based sample of schoolchildren. We observed an inverse association between the consumption of fruit juice, meat and fish and the prevalence of asthma symptoms among children aged 9–11 years. In contrast, fast food consumption and butter intake were associated with an increase prevalence of asthma symptoms among atopic children.

In line with our results, a high intake of fruit juices has been shown to be protective on lung function in subjects aged 11–19 years [33]. Fruits contain antioxidants which are known to play a role on preventing asthma [25], particularly citrus fruits which are rich in vitamin C [25, 33]. In addition, this protective effect of fruit on asthma is also in agreement with other studies done on children [24, 34]. No significant association was found between fruit consumption and BHR, but protective effect has been previously reported [33, 35, 36].

Findings from recent studies suggest that a high intake in omega-3 fatty acids, which are mostly found in fish protects against the development of asthma and atopy in children [26, 37]. Fish is rich in n-3 polyunsaturated fatty acids (PUFA), which are known to have anti-inflammatory effects. Our results are consistent with previous studies that evaluated the association between omega-3 fatty acids and fish intake and asthma related symptoms in children [11, 20, 22, 26]. The protective effect of fish consumption on asthma symptoms is as well in agreement with a report on children in phase II of the ISAAC study [21]. Our observations of no significant association between fish intake and SPT positivity and BHR are consistent with a prospective study done among children aged 5 years [38]. In contrast, the positive association of butter intake with asthma and wheezing is consistent with a study done by Farchi and colleagues in Italy [34]. In our study, the prevalence of asthma symptoms and childhood asthma was also consistent with previous studies [39].

Fast food associated with a higher prevalence of asthma among atopic children is in line with previous research that found that dietary intake of trans-fatty acids which are found in fast food, are positively associated with asthma and atopy [23, 40]. Moreover, canteen lunch, which is commonly known as being a healthy diet for schoolchildren, was negatively associated with asthma symptoms and allergic sensitization, but after stratification by SPT positivity status, positive association was shown between canteen lunch and wheezing among atopic children. Same positive association was found between meat intake and asthma among children with SPT positive. These inverse associations between meat intake, canteen lunch and asthma among children with SPT positive could be explained by the difference of food consumption between atopic and non-atopic children which can differ significantly between them. In addition, meat consumption and eating at the canteen may also vary depending on environmental, economic and social factors which can also explain the fact that we did not had a “dose effect” in our results concerning the protective effect of canteen lunch. Furthermore, we have to consider that not all children eat the same lunches at the canteen which are consisted from different menus containing different food items every day which the child could choose one according to his/her taste.

The strengths of the current study include the large number of participants, its multicenter design and the detailed health outcome assessment including information on atopic sensitization determined by SPT performed in a large number of children aged 9–11 years. Furthermore, the use of the FFQ filled by the children’s parents includes detailed reliable information on the frequency of consumption of food items and beverages because parents are very likely the people the most aware of their children’s diet. Concerning the evaluation of respiratory manifestations, we used internationally validated indicators [28, 41, 42].

The cross-sectional design is a major limitation of the study and it shares the same bias that we found in all observational studies, like a recall bias and not being able to demonstrate causal relationships that could have affected our results. Therefore, these retrospective results need to be confirmed by future prospective studies and/or intervention trials.

The use of the FFQ in this study to assess diet and allergic diseases may be a source of information bias. Information was obtained from children’s parents who could be subject to a recall bias. However, the validity of the FFQ has been proven to be relatively good [43, 44]. Furthermore, the FFQ used in this study do not contain all food items consumed by children, mainly fatty acids consumption, that’s why we analyzed their association to allergic status in a question aside. Also, this FFQ was used in several ISAAC studies but it is not been validated yet in French children aged 9–11 years. Future work will concentrate on the validation of the FFQ in this population. Moreover, we may not have taken all potential confounding variables into consideration, especially physical activity status in children due to the lack of information about it in our data set. However, the multivariate analysis decreases the probability of confounding and an effort was made to correct for gender, current exposure to environmental tobacco smoking (ETS), maternal education, breastfeeding, day care center or nursery, place of residence, number of siblings, overweight and obesity, parental ethnic origins, and parental atopic disease, which are potential confounders [45–48].

An underestimation of prevalence might also affect our results. Asthma and wheezing were reported subjectively by parents as well the identification of tobacco use and smoking. However, the internationally validated indicators we used to evaluate respiratory symptoms decrease the risk of having a differential bias [28, 41, 42].

## Conclusions

In conclusion, dietary factors could be associated with asthma related symptoms and allergic sensitization in children. This study provides further evidence that adherence to a healthy diet that is rich especially in fruits, fish and meat may provide protection against allergies and especially asthma in children. Future prospective and experimental studies are needed to confirm these results so recommendations could be made to children in schools and to their parents, about the importance of adopting a healthy diet to prevent asthma and allergic diseases and about the potential health risks of adopting an unhealthy diet, considering the high prevalence of fast food consumption in children nowadays.

## Abbreviations

ISAAC: International Study of Asthma and Allergies in Childhood
PUFA: Poly unsaturated fatty acids
CNIL: National Commission of Informatics and Civil Liberties
FFQ: Food frequency questionnaire
PEF: Peak expiratory flow
SPT: Skin-prick test
BHR: Bronchial hyper-responsiveness
ETS: Environmental tobacco smoke
m: Mean
SD: Standard deviation
OR: Odds ratio
aOR: Adjusted odds ratio
CI: Confidence interval
SPSS: Statistical Package for Social Sciences.
